# Adaptive Gait Acquisition through Learning Dynamic Stimulus Instinct of Bipedal Robot

**DOI:** 10.3390/biomimetics9060310

**Published:** 2024-05-22

**Authors:** Yuanxi Zhang, Xuechao Chen, Fei Meng, Zhangguo Yu, Yidong Du, Zishun Zhou, Junyao Gao

**Affiliations:** 1 School of Mechatronical Engineering, Beijing Institute of Technology, Beijing 100081, China; zhangyuanxi@bit.edu.cn (Y.Z.); chenxuechao@bit.edu.cn (X.C.); yuzg@bit.edu.cn (Z.Y.); duyidong@bit.edu.cn (Y.D.); zishun.zhou@outlook.com (Z.Z.); gaojunyao@bit.edu.cn (J.G.); 2 Key Laboratory of Biomimetic Robots and Systems, Ministry of Education, Beijing 100081, China

**Keywords:** reinforcement learning, bipedal robot, adaptive locomotion, period dynamic gait

## Abstract

Standard alternating leg motions serve as the foundation for simple bipedal gaits, and the effectiveness of the fixed stimulus signal has been proved in recent studies. However, in order to address perturbations and imbalances, robots require more dynamic gaits. In this paper, we introduce dynamic stimulus signals together with a bipedal locomotion policy into reinforcement learning (RL). Through the learned stimulus frequency policy, we induce the bipedal robot to obtain both three-dimensional (3D) locomotion and an adaptive gait under disturbance without relying on an explicit and model-based gait in both the training stage and deployment. In addition, a set of specialized reward functions focusing on reliable frequency reflections is used in our framework to ensure correspondence between locomotion features and the dynamic stimulus. Moreover, we demonstrate efficient sim-to-real transfer, making a bipedal robot called BITeno achieve robust locomotion and disturbance resistance, even in extreme situations of foot sliding in the real world. In detail, under a sudden change in torso velocity of −1.2 m/s in 0.65 s, the recovery time is within 1.5–2.0 s.

## 1. Introduction

As a type of legged robot, the bipedal robot shows excellent integration with human society. Similarly, environments designed for humans are also suitable for bipedal robots. However, the motion control of legged robots is challenging, especially in tasks with randomness, such as irregular terrain and external disturbances. Until now, equipping a bipedal robot with adaptive gaits has been a complex problem involving rigid body mechanics and actuator control issues.

In previous studies, model-based bipedal locomotion algorithms have made progress [1,2,3]. The simplified mechanical model facilitates bipedal motion planning and balance control, which enables bipedal robots to achieve walking, jogging, and simple jumping in structured environments. However, these bipedal locomotion lack sufficient resistance to non-preset perturbations due to the limitations of artificial state machines, which limits the potential of bipedal robots. Therefore, there is a need to develop more comprehensive and efficient control methods.

As this involves a high-dimensional nonlinear object, the bipedal control problem is well suited for reinforcement learning (RL) methods [4]. On the bipedal platform Cassie [5], some RL methods have made progress [6,7,8,9,10], supporting the importance of electromechanical system design.

In order to simplify the RL training process, some robots utilize a model-based controller for guidance or initialization. Recent RL studies [11,12] have used the residual RL [13,14,15] framework to train corrective policies to track the joint trajectories from the model-based controllers better. However, although the reference trajectories ensure smooth bipedal locomotion, this type of residual RL method sacrifices the advanced knowledge of adaptive gaits. In addition, the framework combining the optimization of a single rigid body model [7] with RL enables the bipedal robot to achieve a maximum speed of 3 m/s, and the footstep-constrained learning [8] can predict the next touchdown location, but the mechanical constraints also limit the RL from exploring bipedal features when trying more dynamic movements. Actually, except for some specific applications of bipedal locomotion, such as using RL to adjust controller parameters [16,17], the model-free RL methods show more potential than model-based ones.

Domain randomization is effective for carrying unsensed dynamic loads [6] and dealing with blind stairs [9], but the essence of this type of method is to expand the knowledge pool of RL without direction. Therefore, it is difficult for users to design skill instructions through such randomized high-dimensional features, which is also the key issue of the model-free RL method. Moreover, RL integrated with imitation learning (IL) can be used to train a more bionic bipedal policy [10,18,19], but the low-dimensional imitation tends to hinder the high-value development of the policy easily. In the RL process, a reasonable expression of bipedal gait is important for the robot to learn robust skills. The parameterized gait library used in [20] preset a locomotion encoder that is beneficial to the RL process, while the learned policy cannot handle situations that are not covered by the gait library. Hence, a training method that is both practicable and explorable will improve the performance of the bipedal locomotion.

In order to learn orderly leg movements, periodic rewards and inputs have been used to provide criteria for training the bipedal policy [21], thereby enabling users to switch between learned gaits. Similarly, a symmetric loss and curriculum learning were designed in [22], and the robot achieved a balanced, low-energy gait. However, since the periodic signals are static for double legs in the frequency domain, it is difficult to train an adaptive gait by only relying on the simple design.

From the perspective of legged robots, the RL method has achieved state-of-the-art results in the field of quadruped robots [23,24,25,26,27]. Quadruped robots have a lower center-of-mass (CoM) height and a larger support area than bipedal robots, which means more stability during the locomotion. The quadruped robot ANYmal [28] utilizes four identical foot trajectory generators (FTGs) [29] together with a neural network policy to learn the dynamic gait to traverse different terrains [25], demonstrating that artificial gaits based on inverse kinematics can assist the quadruped policy in learning skills. Moreover, a more parametric generator based on central pattern generators (CPGs) was used in RL tasks [26] to achieve quadrupedal locomotion on mountain roads. For quadruped robots, regularized FTGs can not only meet the needs of locomotion but also facilitate RL training. But for bipedal robots, the adaptive gaits need to be more dynamic and agile, so neither the generator nor inverse kinematics is helpful for this purpose.

Based on our previous work on the BRS1-P robot [1,30], the 3D locomotion requires an independent state estimation module due to the absence of proprioceptive velocity sensors. As an important observation and reward element, an accurate linear velocity of CoM is the basis of tracking commands. Recently, some model-based algorithms of state estimation were used in RL tasks of bipedal locomotion [10,11,31]. Therefore, an efficient state estimator is necessary for our RL method.

**Figure 1:**
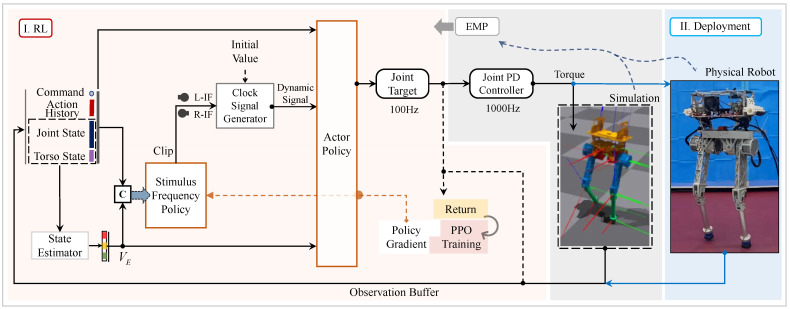
Overview of our RL framework. The learned policies are deployed on the physical robot called BITeno through learning with the embedded mechanics properties (EMP) [30], and the framework consists of two main modules. I. RL: in order to acquire the adaptive gaits, the stimulus frequency policy and the actor policy are trained together. II. Deployment: all of the policies achieve sim-to-real on physical robot BITeno, and the processes corresponding to the dotted lines do not work at this stage.

In this paper, we propose an RL framework consisting of an actor policy and a stimulus policy that outputs dynamic frequencies for the clock signal generator, as shown in Figure 1. Based on the fixed periodic components that are similar to [21] and our previous work [30], we obtained the primary gait in 3D space. In order to design an implicit mechanism that can both correlate adaptive gaits and preserve sufficient exploration potential, we use the dynamic signals as a part of the input of the actor policy. In addition, we introduce a reward component that is corresponding to the stimulus frequency adjustment to train the adaptive gaits.

The contributions of this study can be summarized as a trainable framework, including the gait stimulation policy for RL, which provides both the guidance and exploration space for adaptive gaits. Furthermore, from a bionic perspective, we propose the independent stimulus frequency for each leg to explore a more diverse range of gait patterns. Finally, a series of experiments on physical robots verified the generalization ability of trained policies and demonstrated better anti-disturbance performances than static stimulus methods.

The construction of this paper is as follows. In Section 2, we explain the complete RL framework and details of the BITeno platform. In Section 3, the experimental results and discussions are presented. Finally, in Section 4, we summarize the conclusions of our works in this study.

## 2. Reinforcement Learning Framework and Hardware Platform

We aimed to acquire adaptive gaits using RL methods so that a bipedal robot can resist unknown perturbations while tracking user commands well. In this process, the linear velocity of the CoM is an important observation that cannot be obtained directly by proprioceptive sensors in the physical world. Therefore, we utilize a state estimator based on previous works [23] to map the current state to the linear velocity $V_{E}$, which is considered a cooperator of 3D bipedal locomotion in our methods. In this framework, as shown in Figure 1, two additional agents, namely, the actor policy and stimulus frequency policy, are incorporated into the multilayer perceptron (MLP). In detail, the actor policy operates a core controller outputting the target positions of whole-body joints. Furthermore, the stimulus frequency policy is a front-end, high-dimensional controller that adjusts the left and right implicit frequency (L-IF and R-IF) of two legs according to the real-time states of the robot. More importantly, the clock signal generator was designed to convert the frequency feature into explicit stimulus signals that serve as the key components of the actor policy inputs. Specifically, compared with the locomotion of the quadruped robot, the bipedal locomotion indeed tends to be constrained by the preset gaits like FTGs [25]. And the real-time frequency that was designed for each leg aligns more closely with the bionic principles.

In order to make the RL policies converge well, we trained the robot in simulation as shown in Figure 1. In order to learn a basic balanced skill as a preparation, an initial value was continuously applied to the clock signal generator to output regular signals until the robot acquired a normal gait. During this process, the stimulus frequency policy was trained using supervised learning (SL) according to the initial value, with the goal of enabling the instinctive generation of an original stimulus frequency. Subsequently, both of the policies were trained using RL in simulation. Moreover, for the purpose of reducing syntony and maintaining the control ability, the joint action outputted at 100 Hz was actually the joint reference of the PD controller working at 1000 Hz.

All neural networks in the RL task were trained using the data from the high-performance simulator Isaac Gym [32], and proximal policy optimization [33] was used to train the actor policy and the stimulus frequency policy based on the actor–critic [34] method.

**Figure 2:**
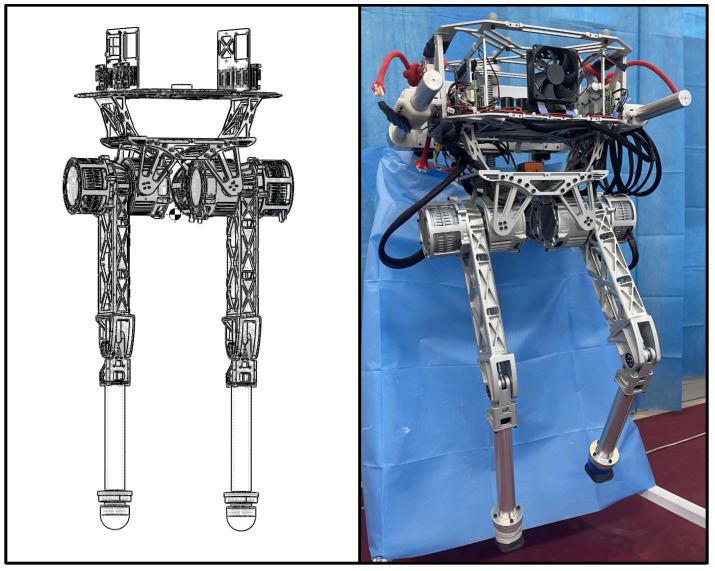
The design of BITeno platform. (**Left**) The mechanical design in simulation, were the feature of each link was assigned according to real materials. (**Right**) The physical robot with an electrical system onboard.

As for the point-footed platform illustrated in Figure 2, the bipedal robot BITeno was originally designed by our team for dynamic locomotion, and the six actuators concern the torque control with a peak value of 62.5 N·m. In addition, the reduction ratio of each joint is 10, which provides abundant torques and enough agility.

The total mass of BITeno is about 16 kg, its standing height is 0.95 m, and the IMU sensor was assembled at the CoM position calculated by the simulator to reduce sim-to-real challenges. In addition, the EtherCAT was used for communication between the computer (ASUS-PN51/R75700U) and joint controllers.

### 2.1. Reinforcement Learning Formulation

The physical world of bipedal locomotion is continuous, but in our RL task, the control problem is formulated in discrete time to simplify the modeling process. At time step *t*, the observation $o_{t}$ represents the state of the current environment, so the locomotion can be explained using a Markov Decision Process (MDP). Each of the MLPs in our RL framework can be regarded as a policy $\pi (a_{t}|o_{t})$ outputting the action $a_{t}$ according to the $o_{t}$, and the environment will move to the next state $o_{t+1}$. In detail, both $a_{t}$ and the transfer of environment come from their respective probability density functions. Furthermore, the reward $R_{t+1}=R\left(o_{t},a_{t},o_{t+1}\right)$ evaluates the control performance of the current unit cycle at time step t+1. However, the scalar reward cannot evaluate the future trend of locomotion, especially on the condition of unknown disturbance. Hence, the expected discounted reward D(π) is introduced in the RL task,

(1) $$D\left(\pi \right)=E\left(\sum_{t=0}^{\infty }\gamma^{t}R_{t}|o_{t},a_{t}\right)$$

and the goal of the RL task is to explore the optimum policy $\pi^{*}\left(A|O\right)$ that is closest to the theoretically ideal policy,

(2) $$\pi^{*}=\underset{\pi }{\arg\max}D\left(\pi \right)$$

where *O* is the observation space, and *A* is the action space. Actually, policies in an RL task can only converge well when the local optimum is covered by *O* and *A*, and the implicit stimulus was designed to suppose this purpose better.

### 2.2. Observation, Action, and Network Architecture

The observations of each policy in our framework are slightly different because of the specific logical relationship between the two policies.

As shown in Table 1, the full observation of the actor policy consists of user command $R^{3}$, including three expected linear velocities along the X, Y, and Z axes, respectively; joint position $R^{6}$; and joint angular velocities $R^{6}$ of 6 actuators, 12 in total; torso pose $R^{3}$ and torso rotational velocities $R^{3}$ obtained by the IMU, six in total; the action history $R^{6}$ of the last time step; estimated linear velocity $R^{3}$; and the dynamic signal $R^{2}$. Moreover, the action of the actor policy is a vector containing the joint target positions $R^{6}$. In addition, the linear velocity vector is concatenated with the observation from proprioceptive sensors, which provides the whole-body feature for the stimulus frequency policy to produce the clipped frequency $R^{2}$ to regulate the dynamic signal.

The policy networks in our work are composed of MLPs. Specifically, the stimulus frequency policy contains two hidden layers with {128, 64} hidden units, and the actor policy has three hidden layers with {512, 256, 128} hidden units. The activation function for each is ReLU.

### 2.3. Clock Signal Generator

The periodic signal is effective guidance for the bipedal gaits [21]. In detail, the frequency, amplitude, and phase variables can influence the joint movement produced by the actor policy; hence, each single leg will reflect the corresponding routine. When two legs work together, an interchanged gait is produced, avoiding the occurrence of asymmetrical and strange gaits in training practice. However, the signals with fixed parameters are still unable to cope with various external disturbances well, especially the fixed frequency. Therefore, the RL-based stimulus frequency policy is proposed to provide dynamic frequencies that are equivalent to the latent feature that is contained by the adaptive gaits.

**Figure 3:**
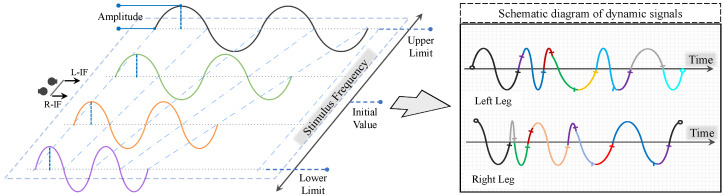
Clock signal generator based on sine (cosine for another leg) function. The clipped output of the stimulus frequency policy is between the lower and upper limit, which ensures the safety of bipedal gaits through a reasonable sine (cosine) stimulus. In the training process and deployment, the dynamic stimulus signal is sampled at 100 Hz, and the colors means signals with different frequencies during sampling. Additionally, the amplitude $A_{p}$ of a signal is a hyper-parameter.

As a source of dynamic signals, the clock signal generator receives the clipped L-IF and R-IF and then produces the dynamic signal for the actor policy. As shown in Figure 3, the real-time signals are concatenated and sampled in a continuous frequency range [2.6π, 3.8π], and the dynamic signal $S_{d}$ is

(3) $$\begin{array}{c} S_{d}\in R^{2}=A_{p}*\left[\sin\left(L-IF*T_{n}\right),\cos\left(R-IF*T_{n}\right)\right] \end{array}$$

(4) $$\begin{array}{c} T_{n}=T_{n-1}+dt \end{array}$$

where $T_{n}$ is the cumulative time of the control process, and dt is 0.001 s. According to this design, the temporal density of the dynamic signals varies with L-IF and R-IF, while the physical time remains uniform. Additionally, the initial value is 3.03 π, which means the desired stepping period is 0.66 s for each leg. Moreover, it should be noted that robots like BITeno require an appropriate stepping frequency to maintain balance because the point-footed design does not support static bipedal locomotion. So frequencies below 2.6 π are not accepted here. Furthermore, the values exceeding the upper limit can easily trigger tremors within the joints, which is obviously detrimental to the adaptive gaits.

### 2.4. Rewards and Training Process

In order to ensure sufficient exploration space, the reward composition based on simplified models and artificial locomotion is ignored in the RL training. After the actor policy acquires basic gait, our framework only focuses on the high-dimensional performance of bipedal locomotion. Therefore, we designed a specialized reward term to induce the L-IF and R-IF. When the robot can perform a stable bipedal gait and resist external disturbances well, it means that the implicit frequency is equipped with an adaptive ability. In addition, as a model-free RL framework, reference trajectories are not involved in the reward functions.

In our framework, the $R_{t}$ is the total reward at time step *t*, and $r_{n}$ is the *n*th-term reward. Each term of the reward functions is weighted by $\beta_{n}$ and represents a certain preference for bipedal locomotion.

(5) $$\begin{array}{c} R_{t}=\sum_{n=0}^{N}\beta_{n}r_{n} \end{array}$$

When the value of $R_{t}$ increases, it is generally considered that the robot’s performance is getting better. Of course, $R_{t}$ just works during training in simulation due to the use of some privileged information (e.g., an accurate torso height). Therefore, the design of the reward functions is an important factor of the sim-to-real transfer, which is also one of the reasons for the existence of $\beta_{n}$. The details of the rewards are in Appendix A.

Since the scale of the data is close to that in our previous work [30], the hyper-parameter of PPO adopted similar settings in this study. As for the training process, a series of external perturbations were applied to the robot at irregular intervals, which allowed both policies to simultaneously acquire more agile skills through interactions with the environment. At the deployment stage, these RL methods also provide sufficient compatibility for the sim-to-real transfer. Additionally, the EMP of the 3D robot was extracted before the RL stage, providing a default simulation setup that follows the features of the physical robot.

## 3. Results and Discussion

**Figure 4:**
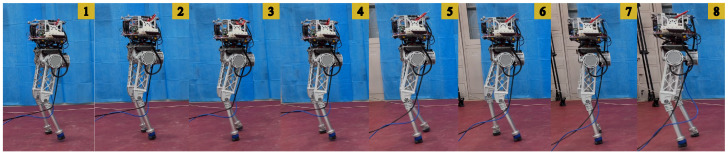
Stable walking (0.65 m/s) gaits. The numbers on the top right represents the time sequences of the locomotion.

**Figure 5:**
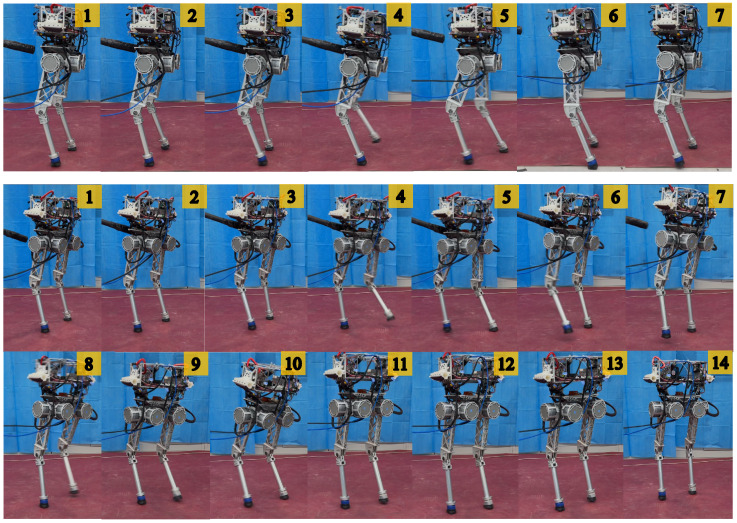
Adaptive resistance gaits under weak and strong disturbances.

The trained policies were successfully deployed on a physical robot using the same framework with the training process, enabling it to achieve impressive 3D bipedal locomotion with BITeno. Through the exploration of RL, BITeno acquired the skill to stably track user commands, as shown in Figure 4. Moreover, under a series of external disturbances, the point-footed BITeno suffered foot slippages during posture adjustment and eventually recovered to a stable gait, demonstrating a robust sim-to-real transfer, as shown in Figure 5.

**Figure 6:**
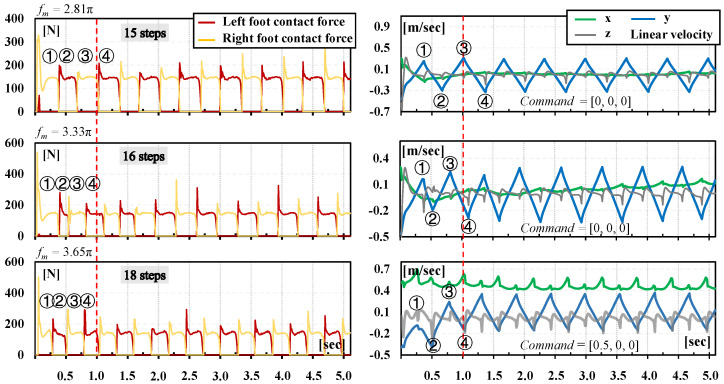
The dynamic step style of the normal gait derived from different stimulus signals. The circled number is the sequence number of sampled touchdown.

In detail, BITeno can implement stable bipedal locomotion using a normal gait on flat ground. Furthermore, different constant frequencies were used as inputs for the actor policy, as shown in Figure 6. Despite no disturbances being applied to the robot, it still generated varying step counts within a fixed period of time. Moreover, the foot contact force and the torso velocity also maintained good coupling over time, which is also a necessary basis for stable gaits. Therefore, all of these results demonstrate the adaptability of the current control framework.

**Figure 7:**
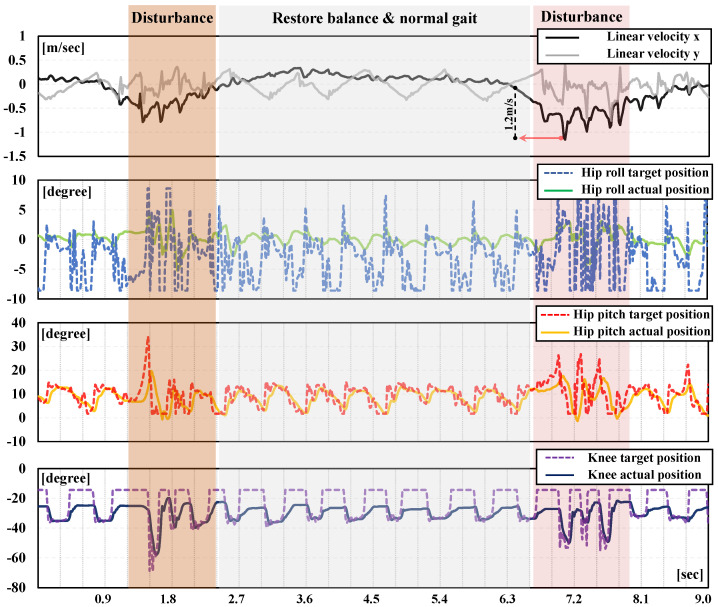
Movement of each joint with dynamic frequency. Under random external disturbances, the entire control policy performs agile resistance, meaning that BITeno acquired adaptive instincts through dynamic stimulus frequencies.

Additionally, because of the effect of the stimulus frequency policy, the actor policy received dynamic signals and adjusted its step frequencies continuously, showcasing versatility in different situations. As for the joint-level movements, all joints consistently maintained a frequency close to the initial value during the locomotion on the flat ground. When faced with sudden changes in the robot status, all joints responded rapidly with a brief frequency adjustment, as shown in Figure 7. Specifically, each joint performed repeated movements consisting of two support phases (or one) and one swing phase (or two) per second for normal gaits. However, in adaptive gaits, the frequency of joint movements increases to a higher level in order to maintain real-time balance.

**Figure 8:**
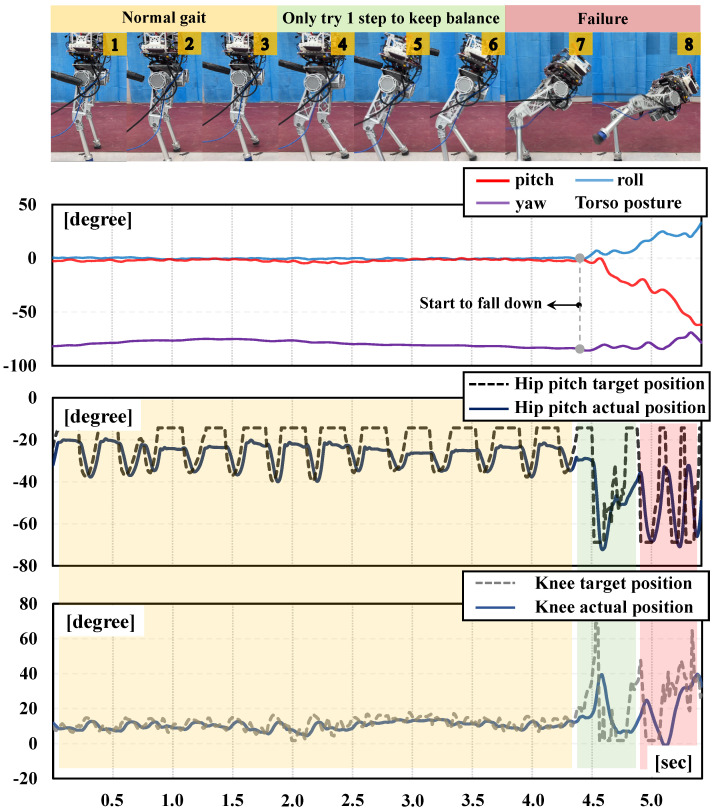
The results of simply using initial values instead of the dynamic stimulus.

As shown in Figure 8, the normal gait of the stimulus frequency policy can achieve primary balance, which verifies the effectiveness of the sim-to-real transfer of our framework on the BITeno hardware platform even using only the static signal. Furthermore, it can be seen from the snapshots in Figure 8 that the robot only made one step as an emergency action under a usual disturbance, resulting in an insufficient dynamic performance of the robot and it falling down. In addition, the support leg should act more agile to maintain balance at this time, but the target positions of the joints did not work with suitable frequencies. Actually, without learning dynamic skills, the bipedal locomotion in this experiment achieved the expected performance and satisfied the upper limit of the capability of the normal gait.

More importantly, concerning the movements of joints shown in Figure 8, the robot did not even show enough struggle action like our full framework after it started to fall down, which further proves the positive impact of learned stimulus signals on adaptive gaits. Through RL training and deployment, we found that normal gait stepping without dynamic stimulation is also relatively stiff, despite that it can remain balanced without disturbance. Additionally, we also found that there is a coupling relationship between the robot’s link size and the natural frequency of the hardware, which is important for further research on our framework.

## 4. Conclusions

Through the methods and experiments presented in this paper, we verified that dynamic clock signals can improve the performance of an RL-learned actor policy. Furthermore, based on the existing gait obtained through a fixed clock, our framework provides more adaptive skills for bipedal robots through learning dynamic stimulus instincts. In detail, the experiments on a physical robot, BITeno, demonstrated both stable walking and adaptive gaits under a series of external disturbances, which also prove that our framework is suitable for sim-to-real transfer. Furthermore, the independent use of the stimulus frequency policy provides a dedicated agent for adaptive gaits, which validates a paradigm for biped robots to learn richer gaits or more complex tasks.

Along this research trajectory, bipedal robots can acquire additional bionic skills through specifically designed agents. In the future, we plan to extend the stimulus frequency policy in this paper to a joint-level dynamic control. Focusing on more bionic designs, we will train the agile locomotion policy to accommodate more complex bipedal tasks through RL methods. As for the natural frequency of the hardware, it is still difficult to achieve accurate calculations based on the rigorous theory of mechanics. Therefore, using it as an implicit feature for the construction of an RL framework can be a part of the research in the future. In addition, the actuator is a key component of the robot joint; hence, seeking a solution with higher accuracy and lower energy loss is another necessary task to acquire more adaptive gaits.

Moreover, the MLP-based policies in this work provide simple learning capabilities. However, for more complex bipedal skills, agents that are more sensitive to time sequences (such as the Transformer [35]) can be used.

## Figures and Tables

**Table 1:** The observation and output of each module.

Data	Dimension	Actor Policy	Stimulus Frequency Policy	State Estimator
Command	3	✔	✔	✗
Joint state	12	✔	✔	✔
Torso state	6	✔	✔	✔
Action history	6	✔	✔	✗
Linear velocity	3	✔	✔	▸
Dynamic signal	2	✔	✗	✗
Joint target	6	▸	✗	✗
Clipped frequency	2	✗	▸	✗

✔ represents the observation, ▸ represents the action, and ✗ means irrelevant data. The joint state is obtained by the off-axis rotary absolute encoder assembled in each joint.

## Data Availability

Data are contained within the article.

## Nomenclature

*q*	Joint position	$q^{*}$	Target joint position
$\overset{..}{q}$	Joint acceleration	τ	Joint torque
$\omega_{torso}$	Angular velocity	${\omega^{*}}_{torso}$	Target angular velocity
$h_{CoM}$	Height	${h^{*}}_{CoM}$	Target height
$v_{CoM}$	Linear velocity	${v^{*}}_{CoM}$	Target linear velocity
roll,pitch,yaw	Poses	$v_{last}$	Last linear velocity
$f_{o}$	Inductive frequency	$f_{m}$	Mean frequency of legs

## Appendix A

### Definition

To train adaptive gaits on basic normal skills, the definition of each reward is as follows. The control period dt is 0.001 s.

Kernel function *a*: $K_{a}$(*x*) = exp $\left(-\frac{{∥x∥}^{2}}{\theta }\right)$, θ = 0.2

Kernel function *b*: $K_{b}$(*x*) = $K_{a}$(*x*)−0.3

Linear velocity reward: $r_{1}$ = $K_{a}\left({v^{*}}_{CoM,xy}-v_{CoM,xy}\right)$dt

Linear velocity penalty: $r_{2}$ = $K_{a}\left({v^{*}}_{CoM,z}-v_{CoM,z}\right)$dt

Angular velocity penalty: $r_{3}$ = $K_{a}\left({\omega^{*}}_{torso}-\omega_{torso}\right)$dt

Pose penalty: $r_{4}$ = ${∥roll∥}^{2}+{∥pitch∥}^{2}+{∥yaw∥}^{2}$dt

Height penalty: $r_{5}$ = ${\left({h^{*}}_{CoM}-h_{CoM}\right)}^{2}$dt

Action rate: $r_{6}$ = $∥a_{t}-a_{t-1}{∥}^{2}$dt

Joint torque penalty: $r_{7}$ = ${∥\tau ∥}^{2}$dt

Joint acceleration penalty: $r_{8}$ = $∥\overset{..}{q}{∥}^{2}$dt

Stimulus induction: $r_{9}$ = $∥v_{CoM}-v_{last}∥$$K_{b}\left(f_{m}-f_{o}\right)$dt,

$f_{o}∼N\left(3.03\pi +\frac{∥v_{CoM}-v_{last}∥}{p},0.25\right)$

Weights: β = {3.0,−1.50,−0.05,−5.0,−1.7,−0.02,

$-5e^{-5},-5e^{-6},5e^{-2}$}

## References

[1] Han L., Chen X., Yu Z., Zhu X., Hashimoto K., Huang Q. (2023). Trajectory-free dynamic locomotion using key trend states for biped robots with point feet. Inf. Sci..

[2] Dong C., Chen X., Yu Z., Liu H., Meng F., Huang Q. (2023). Swift Running Robot Leg: Mechanism Design and Motion-Guided Optimization. IEEE/ASME Trans. Mechatron..

[3] Shigemi S., Goswami A., Vadakkepat P. (2019). ASIMO and Humanoid Robot Research at Honda. Humanoid Robotics: A Reference.

[4] Sutton R.S., Barto A.G. (1998). Reinforcement Learning: An Introduction. IEEE Trans. Neural Netw..

[5] Gong Y., Hartley R., Da X., Hereid A., Harib O., Huang J.K., Grizzle J. Feedback control of a cassie bipedal robot: Walking, standing, and riding a segway. Proceedings of the 2019 American Control Conference (ACC).

[6] Dao J., Green K., Duan H., Fern A., Hurst J. (2022). Sim-to-real learning for bipedal locomotion under unsensed dynamic loads. Proceedings of the 2022 International Conference on Robotics and Automation (ICRA).

[7] Batke R., Yu F., Dao J., Hurst J., Hatton R.L., Fern A., Green K. (2022). Optimizing bipedal maneuvers of single rigid-body models for reinforcement learning. Proceedings of the 2022 IEEE-RAS 21st International Conference on Humanoid Robots (Humanoids).

[8] Duan H., Malik A., Dao J., Saxena A., Green K., Siekmann J., Hurst J. (2022). Sim-to-real learning of footstep-constrained bipedal dynamic walking. Proceedings of the 2022 International Conference on Robotics and Automation (ICRA).

[9] Siekmann J., Green K., Warila J., Fern A., Hurst J. (2021). Blind bipedal stair traversal via sim-to-real reinforcement learning. arXiv.

[10] Li Z., Peng X.B., Abbeel P., Levine S., Berseth G., Sreenath K. (2023). Robust and versatile bipedal jumping control through multi-task reinforcement learning. arXiv.

[11] Duan H., Dao J., Green K., Apgar T., Fern A., Hurst J. (2021). Learning task space actions for bipedal locomotion. Proceedings of the 2021 IEEE International Conference on Robotics and Automation (ICRA).

[12] Siekmann J., Valluri S., Dao J., Bermillo L., Duan H., Fern A., Hurst J. (2020). Learning memory-based control for human-scale bipedal locomotion. arXiv.

[13] Johannink T., Bahl S., Nair A., Luo J., Kumar A., Loskyll M., Ojea J.A., Solowjow E., Levine S. (2019). Residual reinforcement learning for robot control. Proceedings of the 2019 International Conference on Robotics and Automation (ICRA).

[14] Zhang S., Boehmer W., Whiteson S. (2019). Deep residual reinforcement learning. arXiv.

[15] Alakuijala M., Dulac-Arnold G., Mairal J., Ponce J., Schmid C. (2021). Residual reinforcement learning from demonstrations. arXiv.

[16] Csomay-Shanklin N., Tucker M., Dai M., Reher J., Ames A.D. (2022). Learning controller gains on bipedal walking robots via user preferences. Proceedings of the 2022 International Conference on Robotics and Automation (ICRA).

[17] Lenz I., Knepper R.A., Saxena A. (2015). DeepMPC: Learning deep latent features for model predictive control. Robotics: Science and Systems.

[18] Peng X.B., Ma Z., Abbeel P., Levine S., Kanazawa A. (2021). Amp: Adversarial motion priors for stylized physics-based character control. ACM Trans. Graph. ToG.

[19] Vollenweider E., Bjelonic M., Klemm V., Rudin N., Lee J., Hutter M. (2023). Advanced skills through multiple adversarial motion priors in reinforcement learning. Proceedings of the 2023 IEEE International Conference on Robotics and Automation (ICRA).

[20] Li Z., Cheng X., Peng X.B., Abbeel P., Levine S., Berseth G., Sreenath K. (2021). Reinforcement learning for robust parameterized locomotion control of bipedal robots. Proceedings of the 2021 IEEE International Conference on Robotics and Automation (ICRA).

[21] Siekmann J., Godse Y., Fern A., Hurst J. (2021). Sim-to-real learning of all common bipedal gaits via periodic reward composition. Proceedings of the 2021 IEEE International Conference on Robotics and Automation (ICRA).

[22] Yu W., Turk G., Liu C.K. (2018). Learning symmetric and low-energy locomotion. ACM Trans. Graph. TOG.

[23] Bloesch M. (2017). State Estimation for Legged Robots-Kinematics, Inertial Sensing, and Computer Vision. Ph.D. Thesis.

[24] Hwangbo J., Lee J., Dosovitskiy A., Bellicoso D., Tsounis V., Koltun V., Hutter M. (2019). Learning agile and dynamic motor skills for legged robots. Sci. Robot..

[25] Lee J., Hwangbo J., Wellhausen L., Koltun V., Hutter M. (2020). Learning quadrupedal locomotion over challenging terrain. Sci. Robot..

[26] Miki T., Lee J., Hwangbo J., Wellhausen L., Koltun V., Hutter M. (2022). Learning robust perceptive locomotion for quadrupedal robots in the wild. Sci. Robot..

[27] Choi S., Ji G., Park J., Kim H., Mun J., Lee J.H., Hwangbo J. (2023). Learning quadrupedal locomotion on deformable terrain. Sci. Robot..

[28] Hutter M., Gehring C., Jud D., Lauber A., Bellicoso C.D., Tsounis V., Hwangbo J., Bodie K., Fankhauser P., Bloesch M. (2016). Anymal-a highly mobile and dynamic quadrupedal robot. Proceedings of the 2016 IEEE/RSJ International Conference on Intelligent Robots and Systems (IROS).

[29] Iscen A., Caluwaerts K., Tan J., Zhang T., Coumans E., Sindhwani V., Vanhoucke V. Policies modulating trajectory generators. Proceedings of the PMLR: Conference on Robot Learning.

[30] Zhang Y., Chen X., Meng F., Yu Z., Du Y., Gao J., Huang Q. (2024). Learning Robust Locomotion for Bipedal Robot via Embedded Mechanics Properties. J. Bionic Eng..

[31] Xie Z., Clary P., Dao J., Morais P., Hurst J., Panne M. Learning locomotion skills for cassie: Iterative design and sim-to-real. Proceedings of the PMLR: Conference on Robot Learning.

[32] Makoviychuk V., Wawrzyniak L., Guo Y., Lu M., Storey K., Macklin M., Hoeller D., Rudin N., Allshire A., Handa A. (2021). Isaac gym: High performance gpu-based physics simulation for robot learning. arXiv.

[33] Schulman J., Wolski F., Dhariwal P., Radford A., Klimov O. (2017). Proximal policy optimization algorithms. arXiv.

[34] Konda V., Tsitsiklis J. (1999). Actor-critic algorithms. Adv. Neural Inf. Process. Syst..

[35] Vaswani A., Shazeer N., Parmar N., Uszkoreit J., Jones L., Gomez A.N., Polosukhin I. (2017). Attention is all you need. Adv. Neural Inf. Process. Syst..

